# Supplementary material for: Magnetic Resonance Imaging Measurements of the Proximal Palmar Cortex of the Third Metacarpal Bone and the Suspensory Ligament in Non-Lame Endurance Horses before and after Six Months of Training
Source: Animals (Basel). 2023 Mar 20;13(6):1106. doi: 10.3390/ani13061106 (PMC10044202; doi:10.3390/ani13061106)
Supplement: Supplementary file 1 [file animals-13-01106-s001.zip › Questionnaire S1.pdf]

MRI of the proximal metacarpal region of endurance horses before and after 6 months of training and competing – owner questionnaire (pre-season)

Date:

Information about the owner:

Name and surname:

Phone number:

E-mail:

Information about the rider:

Name and surname:

Phone number:

E-mail:

Gender:

Age:

Weight:

longest distance category competed in:

Information about the horse:

Name:

Breed:

Gender:

Age:

Weight (taken at the clinic):

Height:

longest distance category competed in:

Training Practices

1. How long have you owned the horse?

2. When was the last time the horse has entered a race and how long was the race?

    2.1 Did the horse perform according to your expectations?

3. Was the horse rested after the last competition season?

    3.1 How long was the time off training?

    3.2 How long has the horse been back in training now?

*** The following questions are referring to time back in training after the post-season rest.**

4. Is the horse trained exclusively by the rider stated above, or are there also any other people that are involved in the training of the horse on a regular basis?

5. How many times a week is the horse ridden?

6. How many days a week is the horse rested (referring to the days when the horse is not ridden, nor is it lunged, hand walked or put into the walking machine)?

7. How many times a week is the horse ridden in trot?

8. How many times a week is the horse ridden in canter?

9. How long is the longest training session in a week (how many km)?

10. What type of terrain is the horse usually trained on? (please thick the appropriate box/boxes)

- Flat
- Undulating
- Hilly

- Hard (like a gravel road or an asphalt road)
- Soft (like a filed or a forest road)
- Deep (like in a riding arena filled with sand or wood shavings)

- Other (please specify): ______________________________________

11. Is the horse ever trained on asphalt?

11.1 What is the fastest gait in which the horse is trained on asphalt?

12. Is the horse trained in any other discipline?

13. Is any unridden work also a part of the training routine for this horse? (please thick the appropriate box/boxes)

- Lunging
- Hand walking
- Time spend in the walking machine
- None of the above
- Other (please specify): _________________________

14. How satisfied are you with the horse's progress in training?

14.1 Did you recently notice any of the following during your training sessions? (please thick the appropriate box/boxes)

- The horse is reluctant to go forward and/or backward.
- The horse is reluctant to bend and/or leans on the rider's hands.
- The horse lacks connection to the bit.
- The horse lacks the hind leg engagement and/or impulsion.
- The horse feels stiff.
- When lunged the horse turns the body inwards and looks to the outside.

15. How often is the horse re-shoed/re-trimmed?

15.1 What type of shoes are you currently using with this horse?

16. Has the horse been saddle fitted recently?

16.1 How long has it been since?

Health survey

1. Has the horse ever suffered from lameness or back pain?

   1.1 When was the last episode?

2. Was the horse ever diagnosed or treated by a veterinary surgeon for tendon or ligament injury, foot pain, joint pain, or thoracolumbar region pain?

3. Did you recently notice any swelling, heath, or painful response to touch in cannon region of the forelimbs?

4. Did you recently observe any signs of trauma in the cannon region of the forelimbs (scratches, cuts, abrasions etc.)?

4. Was the horse ever eliminated from the race due to veterinary reasons?

   4.1. What were the reasons behind it?

5. Has the horse ever been sedated?

6.Has the horse had any reaction to any medication ?
