# Supplementary material for: Magnetic Resonance Imaging Measurements of the Proximal Palmar Cortex of the Third Metacarpal Bone and the Suspensory Ligament in Non-Lame Endurance Horses before and after Six Months of Training
Source: Animals (Basel). 2023 Mar 20;13(6):1106. doi: 10.3390/ani13061106 (PMC10044202; doi:10.3390/ani13061106)
Supplement: Supplementary file 1 [file animals-13-01106-s001.zip › TABLE S1_before Table S2_09.03.2023.pdf]

Table S1. Results of the independent samples t-test comparing the thickness of the palmar cortex of the third metacarpal bone (PcMcIII) between novice (n=6) and experienced (n = 6) endurance horses at 2, 3 5 and 7 cm distal to the carpometacarpal joint (CMCJ); 25%, 50%, 75% of the mediolateral width of the of the PcMIII, pre-season = 1st examination (n = 12), post-season = 2nd examination (n = 11), SD – standard deviation; MD – mean difference; CI– confidence intervals. * = statistically significant, $p < 0.05$.

	Novice		Experienced		MD [mm]	p value	95% CI [mm]
	Mean [mm]	SD [mm]	Mean [mm]	SD [mm]			
**Pre-season**							
2 cm distal to CMCJ							
25% PcMcIII	2.7	0.8	4.0	1.1	1.2	<0.01*	0.5-2.0
50 % PcMcIII	2.5	0.9	3.7	1.1	1.1	<0.01*	0.3, 2.0
75% PcMcIII	2.5	0.8	2.7	0.9	0.2	0.48	-0.5, 1.0
3 cm distal to CMCJ							
25% PcMcIII	3.9	1.1	5.1	0.6	1.2	<0.01*	0.4, 1.9
50 % PcMcIII	3.8	1.0	4.8	0.9	1.0	0.02*	0.1, 1.8
75% PcMcIII	3.6	0.8	3.5	0.5	-0.1	0.68	-0.7, 0.5
5 cm distal to CMCJ							
25% PcMcIII	4.5	1.2	5.2	0.9	0.7	0.14	-0.2, 1.6
50 % PcMcIII	4.3	1.1	4.6	0.8	0.3	0.42	0.0, 1.1
75% PcMcIII	4.3	0.9	4.4	1.0	0.1	0.70	-0.7, 1.0
7cm distal to CMCJ							
25% PcMcIII	5.7	1.3	5.7	1.2	0.0	0.94	-1.0, 1.1
50 % PcMcIII	5.3	1.3	5.4	1.1	0.0	0.95	-1.0, 1.0
75% PcMcIII	5.5	1.2	5.4	1.2	-0.2	0.73	-1.2, 0.8
**Post-season**							
2 cm distal to CMCJ							
25% PcMcIII	3.1	0.7	4.0	0.8	0.9	0.01*	0.3, 1.6
50 % PcMcIII	3.0	0.9	3.5	1.1	0.6	0.18	-0.3, 1.5
75% PcMcIII	2.5	0.6	3.0	0.9	0.5	0.14	-0.2, 1.2
3 cm distal to CMCJ							
25% PcMcIII	3.5	1.0	4.8	0.8	1.4	<0.01*	0.6, 2.2
50 % PcMcIII	3.5	0.8	4.6	0.8	1.2	<0.01*	0.4, 1.9
75% PcMcIII	3.1	0.8	3.4	0.9	0.3	0.43	-0.5, 1.1
5 cm distal to CMCJ							
25% PcMcIII	4.5	0.8	4.7	1.1	0.2	0.67	-0.6, 1.0
50 % PcMcIII	4.2	0.8	4.4	1.1	0.1	0.75	-0.7, 1.0
75% PcMcIII	4.4	0.6	4.3	0.9	0.0	0.93	-0.7, 0.64
7 cm distal to CMCJ							
25% PcMcIII	5.3	1.2	5.1	1.6	-0.2	0.71	-1.5, 1.0
50 % PcMcIII	5.2	1.0	4.8	1.6	-0.3	0.55	-1.5, 0.8
75% PcMcIII	5.3	0.7	4.7	1.5	-0.6	0.30	-1.7, 0.5
